# Influence of Tobacco Marketing on Nepalese Adolescents: Cigarette Use and Susceptibility to Cigarette Use

**DOI:** 10.31557/APJCP.2020.21.9.2689

**Published:** 2020-09

**Authors:** Prem Gautam, Dharma Bhatta, Eva Sharma, Abir Rahman, Rahel Dawit, Wei Li, Mohammad Ebrahimi Kalan, Srijana Acharya Gautam, Tan Li, Wasim Maziak

**Affiliations:** 1 *Department of Epidemiology, Florida International University, USA. *; 2 *Center for Tobacco Control Research and Education, University of California, San Francisco, California, USA. *; 3 *Global Cancer Program, Helen Diller Family Comprehensive Cancer Center, University of California, San Francisco, CA, USA. *; 4 *Westat, 1600 Research Boulevard, Rockville, MD 20850, USA. *; 5 *Ace Institute of Management, Pokhara University, Kathmandu, Nepal. *; 6 *Department of Biostatistics, Robert Stempel College of Public Health and Social Work, Florida International University, Miami, Florida, USA. *; 7 *Syrian Center for Tobacco Studies, Aleppo, Syrian Arab Republic. *

**Keywords:** Tobacco marketing, cigarette use, susceptibility, adolescents, epidemiology

## Abstract

**Background:**

Tobacco advertising, promotion, and sponsorship (TAPS) are common tactics of the tobacco industry to encourage adolescents to use tobacco products.

**Objective:**

The objective of the study is to assess the influence of TAPS on cigarette use and susceptibility to cigarette use among Nepalese adolescents.

**Materials and Methods:**

Data (n=2,878) were drawn from the Global Youth Tobacco Survey for Nepal (GYTS, 2011). Channel-specific and cumulative TAPS exposure were the primary exposures of the study. Six multivariate logistic regression analyses were performed to examine current and ever cigarette use outcome with exposure to TAPS. Six binary logistic regression analyses were applied to determine the susceptibility to cigarette use when exposed to TAPS.

**Results:**

Channel-specific TAPS analyses show that indirect TAPS increases the odds of all the three outcomes; current cigarette use (OR=1.68, 95% CI=1.10-2.58), ever cigarette use (OR=1.81, 95% CI=1.23-2.65) and susceptibility to cigarette use (OR=1.65, 95% CI=1.25-2.19) after adjusting for the covariates. Television (TV) and movies exposure decreases the odds of susceptibility to cigarette use (OR=0.55, 95% CI=0.31-0.97). Cumulative TAPS analyses show that exposure to 5 sources of TAPS increases the odds of current cigarette use (OR=2.53, 95% CI=1.21-5.29). Being male increases the odds of all the three outcomes; current (OR=3.52, 95% CI=2.11-5.87), ever (OR=2.51, 95% CI=1.69-3.73) and susceptibility to cigarette use (OR=1.31, 95% CI=1.01-1.69). Social influence is likely to increase current (OR=6.47, 95% CI=2.50-16.74), ever (OR=1.79, 95% CI=1.10-2.93) and susceptibility to cigarette use (OR=1.66, 95% CI=1.25-2.21).

**Conclusion:**

Indirect TAPS exposure increased the current, ever, and susceptibility to cigarette use among Nepalese adolescents. Overall, the current use of cigarettes followed a dose-response relationship with TAPS exposure. The result implies a requirement of active surveillance of tobacco products and future research on adolescent-focused tobacco marketing in Nepal.

## Introduction

Every year more than 8 million people die from tobacco use globally (WHO, 2019a). A systematic analysis of data from 195 countries and territories shows that 6.4 million (11.5%) of global deaths in 2015 were attributable to tobacco smoking (Collaborators, 2017). Most of the 1.1 billion smokers worldwide live in low- and middle-income countries that have a huge potential for the tobacco industry (TI) marketing (WHO, 2019a). Historically, TIs have been known to target the young population, sell at low prices, flaunt tobacco control policies, and lobby aggressively against the development of new policies to increase their sales (Gilmore et al., 2015). For example, in Namibia, the delay in passing the Tobacco Products Control Act by nearly 20 years is mostly attributed to TI interference (Tam and Van, 2014). 

Most addicted adult smokers begin their perilous journey with tobacco during adolescence providing lifelong customers for the TIs (US Department of Health and Human Services, 2012). According to the Tobacco Atlas (2019), among adolescents ages 13-15 years worldwide, approximately 25 million boys and 13 million girls use cigarettes or smokeless tobacco products (The Tobacco Atlas, 2019). Young people represent a marketing goldmine for the TIs and need to be a cornerstone in tobacco control efforts aiming at reducing the health burden of tobacco use.

In the context of Nepal, tobacco smoking leads to more than 27,000 deaths annually (The Tobacco Atlas, 2019). Cigarette smoking is the major form of tobacco use in Nepal along with hand-rolled cigarettes (bidis) (Shrestha et al., 2019). Data from the Global School-Based Student Health Survey Nepal (2015) shows that 6.4% of adolescents (7th to 11th grades) currently smoke cigarettes (Nepal Health Research Council, 2017). Studies conducted in different cities of Nepal revealed that most of the students initiate smoking between the ages of 13-16 years and the initiation age range from 5-18 years (Paudel, 2003; Sreeramareddy et al., 2008). The earlier they start, the higher the risk for daily, heavy smoking leading to nicotine dependence and difficulty quitting in future, which ultimately increases the risk of tobacco-induced diseases such as coronary heart disease, cancer, and chronic obstructive pulmonary disease (COPD) (Sylvestre et al., 2018; US Department of Health and Human Services, 2014).

In response to the global tobacco epidemic, a global initiative represented by the WHO Framework Convention on Tobacco Control (WHO FCTC) treaty was initiated in 2003 (WHO, 2003). Nepal signed the WHO FCTC in 2003 and ratified it in 2006 (WHO, 2019b). Additionally, Nepal passed the Tobacco Product (Control and Regulation) Act of 2011 requiring a complete ban on advertisement, promotion and sponsorship (TAPS) of all tobacco products; (cigarettes, bidi, and smokeless tobacco) among other measures (Campaign for Tobacco-Free Kids, 2019; WHO, 2012). Despite these efforts, the data from three consecutive Demographic Health Surveys (DHS, 2006-2016) shows an increase in tobacco smoking (~12% to 16%) and smokeless tobacco use (~12% to 17%) among males of age less than 20 years during the period (Shrestha et al., 2019). Given the high likelihood of under-reporting of smoking among Nepalese women, the increasing prevalence of smoking among young male highlights weakness in existing tobacco regulatory programs of Nepal and their inability to reach out to the group that comprises of 22% (6.38 million) of Nepal’s population (Bulletin of the WHO, 2017; Shrestha et al., 2019). The increasing prevalence also indicates that the TIs have sufficiently been able to counter the tobacco control efforts and take advantage of weak enforcement (Bhatta et al., 2019a). In 2017, Nepal’s biggest tobacco company “Surya Nepal Pvt. Ltd” spent Nepalese Rupees 12,546,009 (approximately 125,460 USD) on promotion and sponsorship of their products (Surya Nepal Private Nepal, 2017). The WHO tobacco assessment report (2012) highlighted a weakness of the Nepalese government to restrict the advertisements from international TV and radio stations (WHO, 2012). Studies show that exposure to cigarette advertisements leads to smoking initiation and regular smoking among adolescents (Hanewinkel et al., 2011; Henriksen et al., 2010; Slater et al., 2007). To date, no study has examined the effects of TAPS on cigarette smoking among Nepalese adolescents. The objective of the study is to examine the effects of TAPS on cigarette use and susceptibility to cigarette use among Nepalese adolescents. Examining the TAPS effect using nationally representative data will help guide complete enforcement of the policy to curb tobacco use in the population. 

## Materials and Methods

*Data Source and Study population*

Participants’ TAPS exposure, cigarette use, and susceptibility to cigarette use data were obtained from the most recent publicly available GYTS (2011) for Nepal which includes 2,878 adolescents. GYTS is a school-based survey among students between the ages of 13-15 years. It includes a standardized questionnaire to assess tobacco use, perceptions, and knowledge about harmful effects, tobacco advertisements, and parental and peer influence. The multistage sampling design was used to produce a nationally representative sample (Nepal-GYTS 2011, 2019). 

*Outcome variables*

Cigarette use: In line with previous research (Chido-Amajuoyi et al., 2017), adolescents who reported “one or more days” to the question “During the past 30 days, on how many days did you smoke cigarettes?” were considered as the current users. Those who reported “Yes” to the question “Have you ever tried or experimented with cigarette smoking, even one or two puffs?” but “No” to the question “During the past 30 days, on how many days did you smoke cigarettes?” were considered as ever users. Those who reported “No” to the question “Have you ever tried or experimented with cigarette smoking, even one or two puffs?” were considered as never users.

Susceptibility to cigarette use: Never users were further asked two questions: “If one of your best friends gives you a cigarette, would you smoke it?” and “At any time during the next 12 months do you think you will smoke a cigarette?”. Response options for these two questions were “definitely yes,” “probably yes,” “probably not,” and “definitely not”. Only students who had never used cigarettes and answered “definitely no” for both questions were considered as non-susceptible and the rest were classified as susceptible to using cigarettes.

*Independent variables*

The main predictor variable for the study was exposure to TAPS which was categorized into channel-specific and cumulative-exposure. The channel-specific exposure was categorized as follows:

a) Indirect TAPS: adolescents who responded “yes” to either of the following questions: “has a cigarette representative ever offered you a free cigarette” and “do you have something (t-shirt, pen, backpack, etc.) with a cigarette brand logo on it?” were considered as indirect TAPS.

b) TV and movies: adolescents who reported “sometimes/a few” and “a lot” to the questions: (1)“during the past 30 days, when you watched sports events or other programs on TV, how often did you see cigarette brand names?” and (2) “when watching TV, videos, or movies, how often do you see actors smoking?” were considered as TV and movies.

c) Sponsored events: adolescents who reported “sometimes/a few” and “a lot” to the question “when you go to sports events, tradeshows, concerts or community events, how often do you see advertisements for cigarettes” were considered as sponsored events.

d) Print media: adolescents who reported “sometimes/a few” and “a lot” to the question “during the past 30 days, how many advertisements or promotions for cigarettes have you seen in newspapers or magazines?” were considered as print media. 

e) Billboards: adolescents who reported “sometimes/a few” and “a lot” to the question 

 “during the past 30 days, how many advertisements for cigarettes have you seen on billboards?” were considered as billboards. 

The cumulative-exposure variable was created by summing the number of channel-specific exposures. It was categorized into two or less, three sources, four sources, and five sources.

*Covariates*

Sociodemographic factors and current use of other tobacco products were included as covariates for the study. Gender was categorized into male and female. Age was categorized into three groups (≤11 years, 12-16 years, and ≥17 years). Grade level was categorized into four classes (7, 8, 9 and 10). Social influence was assessed based on the response “Yes” to either of the questions (1) “Do your parents smoke?” and (2) “Do any of your closet friends smoke cigarettes?”. Other tobacco product use was assessed based on the response “Yes” to the question “During the past 30 days (one month), did you smoke any form of tobacco products other than cigarettes or bidis (e.g. chilim, hukkah, cigars, cigarillos, little cigars, pipes)?”.

*Statistical analysis*

Differences between adolescents’ cigarette use behaviors and their characteristics were examined using the Chi-square test. Six multivariate logistic regression analyses were conducted to assess current, ever, and never smoking outcomes (five for channel-specific TAPS and one for cumulative TAPS) controlling for gender, age, grade level, social influence, and other tobacco products use. Six binary logistic regression analyses were performed to assess the susceptibility of cigarette use (five for channel-specific TAPS and one for cumulative TAPS) controlling for gender, age, grade, social influence, and other tobacco products use. Adjusted odds ratios (aORs) and corresponding 95% confidence interval (95% CI) were reported. P-value <0.05 was considered statistically significant. The variable ‘FinalWgt’ was used to get weighted outcomes. All the analyses were conducted using SAS version 9.4 (SAS Institute Inc., Cary, North Carolina).

## Results

*Descriptive statistics*

Among 2,878 participants, 1,398 (50.4%) were female and 1,378 (49.6%) were male. The majority (86.40%) of the participants were between the ages of 12-16 years. The prevalence of current smoking was 5.03%, ever smoking was 5.66% and never smoking was 89.32%. About seventeen percent (17.25%) of the participants were susceptible to cigarette smoking. Exposure to TV and movies (96.09%) was the most prevalent source of channel-specific TAPS exposure. Exposure to four sources of TAPS (41.89%) was the most prevalent cumulative TAPS exposure. The past 30-day use of other tobacco products among the participants was 6.06%. About seventy-two percent (71.66%) of the participants reported that their family or friends smoked cigarettes. A detailed description of the participants’ smoking characteristics is shown in Table 1 and the susceptibility characteristics are shown in Table 2. 

*Current cigarette use*

Channel-specific TAPS analyses show that the current use of cigarettes increased with indirect TAPS (OR=1.68, 95% CI=1.10-2.58) after adjusting for covariates (Table 3).

Cumulative TAPS analysis shows that current use of cigarette increased with 5 sources of TAPS exposure (OR=2.53, 95% CI=1.21-5.29), male (OR=3.52, 95% CI=2.11-5.87), grade level 8 (OR=4.60, 95% CI=1.88-11.27), grade level 10 (OR=5.55, 95% CI=2.18-14.18), social influence (OR=6.47, 95% CI=2.50-16.74) and other tobacco use (OR=4.12, 95% CI=2.32-7.31) (Table 3). 

*Ever cigarette use*

During channel-specific TAPS analyses, ever cigarette use increased with exposure to indirect TAPS (OR=1.81, 95% CI=1.23-2.65) after adjusting for covariates (Table 3). 

During cumulative TAPS analysis, ever cigarette use increased among male (OR=2.51, 95% CI=1.69-3.73), grade level 10 (OR=2.64, 95% CI=1.50-4.64), social influence (OR=1.79, 95% CI=1.10-2.93) and other tobacco use (OR=4.04, 95% CI=2.29-7.13). Ever cigarette use decreased among age group 12-16 years (OR=0.36, 95% CI=0.17-0.75) (Table 3). 

*Susceptibility to cigarette use*

Susceptibility to cigarette use was higher with exposure to indirect TAPS (OR=1.65, 95% CI=1.25-2.19) and lower with TV and movies (OR=0.55, 95% CI=0.31-0.97) after adjusting for covariates during channel-specific TAPS analyses (Table 4). 

With cumulative TAPS analysis, susceptibility to cigarette use was higher among male (OR=1.31, 95% CI=1.01-1.69) and those who have social influence (OR=1.66, 95% CI=1.25-2.21) (Table 4).

**Table 1:** Descriptive Smoking cCharacteristics of the Nepalese Adolescents, GYTS 2011 (n=2,705)

Variables	Current user, n (%)	Ever user*, n (%)	Never user, n (%)	p-value
Prevalence	136 (5.03)	153 (5.66)	2416 (89.32)	
Channel-specific TAPS				
Indirect TAPS				
Yes	55 (41.04)	58 (38.16)	551 (22.90)	<0.001
No	79 (58.96)	94 (61.84)	1855 (77.10)	
TV and movies				
Yes	134 (99.26)	133 (97.08)	2173 (96.19)	0.162
No	1 (0.74)	4 (2.92)	86 (3.81)	
Sponsored events				
Yes	96 (76.80)	99 (80.49)	1622 (77.09)	0.678
No	29 (23.20)	24 (19.51)	482 (22.91)	
Print media				
Yes	108 (80.60)	117 (78.00)	1813 (75.92)	0.408
No	26 (19.40)	33 (22.00)	575 (24.08)	
Billboards				
Yes	119 (88.15)	126 (84.00)	1915 (79.82)	0.032
No	16 (11.85)	24 (16.00)	484 (20.18)	
Cumulative TAPS				
≤2 sources	15 (11.03)	24 (15.76)	480 (19.89)	<0.001
3 sources	33 (24.26)	41 (26.97)	625 (25.90)	
4 sources	54 (39.71)	63 (41.45)	1038 (43.02)	
5 sources	34 (25.00)	24 (15.79)	270 (11.19)	
Covariates				
Gender				
Male	103 (79.84)	107 (72.79)	1094 (41.76)	<0.001
Female	26 (20.16)	40 (27.21)	1250 (53.33)	
Age				
≤11	5 (3.76)	11 (7.38)	80 (3.39)	<0.001
12-16	100 (75.19)	121 (81.21)	2084 (88.42)	
≥17	28 (21.05)	17 (11.41)	193 (8.19)	
Grade level				
7	8 (30.53)	27 (17.88)	664 (28.11)	<0.001
8	40 (30.53)	36 (23.84)	544 (23.03)	
9	48 (36.64)	49 (32.45)	791 (33.49)	
10	35 (26.72)	39 (25.83)	363 (15.37)	
Social influence				
Yes	131 (96.32)	128 (83.66)	1669 (69.08)	<0.001
No	5 (3.68)	25 (16.34)	747 (30.92)	
Other tobacco product use				
Yes	28 (21.05)	23 (15.23)	98 (4.10)	<0.001
No	105 (78.95)	128 (84.77)	2294 (78.95)	

*Ever user excludes current users.

**Table 2:** Descriptive Smoking-Susceptibility Characteristics of the Nepalese Adolescents (n=2,411)

Variables	Susceptible, n (%)	Non-susceptible, n (%)	p-value
Prevalence	416 (17.25)	1995 (82.75)	
Channel-specific TAPS			
Indirect TAPS			
Yes	134 (32.45)	412 (20.72)	<0.001
No	279 (67.55)	1576 (79.28)	
TV and movies			
Yes	364 (94.79)	1805 (96.52)	0.105
No	20 (5.21)	66 (3.48)	
Sponsored events			
Yes	277 (77.81)	1344 (76.98)	0.733
No	79 (22.19)	402 (23.02)	
Print media			
Yes	316 (77.07)	1494 (75.68)	0.55
No	94 (22.93)	480 (24.32)	
Billboards			
Yes	333 (80.05)	1578 (79.78)	0.901
No	83 (19.95)	400 (20.22)	
Cumulative TAPS			
≤2 sources	79 (18.99)	400 (20.08)	0.011
3 sources	99 (23.80)	525 (26.36)	
4 sources	172 (41.35)	863 (43.32)	
5 sources	66 (15.87)	204 (10.24)	
Covariates			
Gender			
Male	216 (53.87)	875 (45.13)	0.001
Female	185 (46.13)	1064 (54.87)	
Age			
≤11	20 (4.90)	60 (3.09)	0.011
12-16	343 (84.07)	1736 (89.30)	
≥17	45 (11.03)	148 (7.61)	
Grade level			
7	94 (23.15)	566 (29.01)	0.106
8	100 (24.63)	444 (22.76)	
9	149 (36.70)	641 (32.85)	
10	63 (15.52)	300 (15.38)	
Social influence			
Yes	325 (78.13)	1342 (67.27)	<0.001
No	91 (21.88)	653 (32.73)	
Other tobacco product use			
Yes	26 (6.42)	72 (3.63)	0.01
No	379 (93.58)	1911 (96.37)	

**Table 3:** Association between Cigarette Use and Exposure to TAPS among the Nepalese Adolescents

Variables	Current vs. Never user	Ever vs. Never user
	OR (95% CI)	OR (95% CI)
Channel-specific (TAPS)		
Indirect TAPS	**1.68 (1.10-2.58) ** ^a^	**1.81 (1.23-2.65) ** ^a^
TV and movies	5.15 (0.69-38.45) ^a^	0.76 (0.24-2.34) ^a^
Sponsored events	0.92 (0.57-1.48) ^a^	1.13 (0.67-1.90) ^a^
Print media	1.23 (0.75-2.04) ^a^	1.05 (0.67-1.65) ^a^
Billboards	1.69 (0.86-3.29) ^a^	1.07 (0.66-1.74) ^a^
Cumulative TAPS		
≤2 sources	Ref.	Ref.
3 sources	1.28 (0.61-2.66)	1.00 (0.56-1.78)
4 sources	1.45 (0.73-2.90)	0.97 (0.57-1.65)
5 sources	**2.53 (1.21-5.29)**	1.23 (0.64-2.36)
Male (ref=female)	**3.52 (2.11-5.87)**	**2.51 (1.69-3.73)**
Age		
≤11 years	Ref.	Ref.
12-16 years	0.82 (0.25-2.62)	**0.36 (0.17-0.75)**
≥17 years	1.35 (0.39-4.67)	0.34 (0.14-0.85)
Grade level		
7	Ref.	Ref.
8	**4.60 (1.88-11.27)**	1.66 (0.94-2.91)
9	3.28 (1.35-7.98)	1.63 (0.94-2.84)
10	**5.55 (2.18-14.18)**	**2.64 (1.50-4.64)**
Social influence	**6.47 (2.50-16.74)**	**1.79 (1.10-2.93)**
Other tobacco product use	**4.12 (2.32-7.31)**	**4.04 (2.29-7.13)**

^a ^Odds ratios were adjusted for gender, age, grade level, social influence and past-30 days use of other tobacco products; Bold numbers indicate statistical significance at p<0.05.

**Table 4:** Association between Cigarette Use Susceptibility and TAPS Exposure among the Nepalese Adolescents

Variables	Susceptibility OR (95% CI)
Channel-specific (TAPS)	
Indirect TAPS	**1.65 (1.25-2.19) ** ^a^
TV and movies	**0.55 (0.31-0.97) ** ^a^
Sponsored events	1.00 (0.73-1.35) ^a^
Print media	1.14 (0.85-1.51) ^a^
Billboards	1.09 (0.81-1.48) ^a^
Cumulative TAPS	
2 sources	Ref.
3 sources	1.00 (0.69-1.45)
4 sources	1.00 (0.72-1.41)
5 sources	1.37 (0.90-2.06)
Male (ref=female)	**1.31 (1.01-1.69)**
Age	
≤11 years	Ref.
12-16 years	0.74 (0.40-1.37)
≥17 years	1.05 (0.49-2.23)
Grade level	
7	Ref.
8	1.22 (0.86-1.71)
9	1.47 (1.06-2.03)
10	1.50 (0.98—2.31)
Social influence	**1.66 (1.25-2.21)**
Other tobacco product use	1.58 (0.92-2.70)

^a^Odds ratios were adjusted for gender, age, grade level, social influence and past-30 days use of other tobacco products; Bold numbers indicate statistical significance at p<0.05.

## Discussion

This study is the first to examine the effects of tobacco marketing on Nepalese adolescents’ cigarette use behaviors and susceptibility to cigarette use. Our findings show a significant association between the channel-specific and cumulative TAPS exposure, and the adolescents’ cigarette use behavior. Overall, the odds of cigarette use followed a pattern of smoking where current users had a higher odds ratio than ever users compared to never users. The overall odds of the current cigarette use followed a dose-response relationship with the TAPS, where cumulative exposure of 5 TAPS sources had the highest odds ratio compared to 2 or lesser sources. These results indicate that tobacco marketing in Nepal has a significant effect on Nepalese adolescents’ cigarette use behavior which highlights a need for further monitoring and research.

Our findings are in accord with previous studies conducted in Nigeria (Chido-Amajuoyi et al., 2017), the United States (Altman et al., 1996; Evans et al., 1995; Pierce et al., 1998, US Department of Health and Human Services, 2014), Zambia (Zulu et al., 2009) and a Cochrane systematic review assessing the impact of tobacco advertisement and promotion among adolescents (Lovato et al., 2003). A higher risk of smoking among adolescents was similar to that of Spanish children of similar age (Lopez et al., 2004). The higher odds of all three outcomes with indirect TAPS exposure may be due to its ability to circumvent the existing ban (Chido-Amajuoyi et al., 2017). The aggressive branding and corporate sponsorship of TIs in sports and musical programs that attract a large number of adolescents might be some other reasons for the higher odds (Shrestha et al., 2019). TV and movie exposure was negatively associated with the susceptibility of smoking among never smokers. This might be related to several factors such as strong determination of not smoking among never-smokers or may be increased in awareness through TV programs or maybe due to lack of access to TV (Wakefield et al., 2003; Khanal et al., 2013). 

Similar to a study conducted among Polish adolescents aged 13-19 years, we found a higher cigarette use prevalence and more susceptibility to use cigarettes among boys compared to girls (Polanska et al., 2016). In the context of Nepalese society, smoking among males is considered an acceptable behavior but not for female and there is a higher chance of under-reporting of tobacco smoking among females (Shrestha et al., 2019). Contrary to the Polish study, we found an increase in cigarette use with an overall increase in school grades (Polanska et al., 2016). This could be due to a greater influence on risk-taking behaviors among adolescents. A study shows that adolescents underestimate the personal risks and risk of smoking and are confident that they can quit before being addicted to or before their health is affected by smoking (Weinstein et al., 1998).

The use of other tobacco products in past-30 days among adolescents was found to be associated with the increased likelihood of cigarette use. This is in line with a cross-sectional study among US adults showing a positive relation between cigarette smoking and concurrent use of other tobacco products (dual or poly tobacco use), especially in males compared to females (Backinger et al., 2008). Social influence played a significant effect on all three outcomes. This might be explained by social learning theory; new behaviors can be learned by imitating and observing others (Kobus, 2003). Studies among adolescents of age range 11-19 years old show that those who have a higher number of family members and peer smokers are more likely to use and are more susceptible to smoke in the future (Polanska et al., 2016; Scalici and Schulz, 2017). 

The study has several limitations. First, data for the study was collected in 2011 (the most recent GYTS data for Nepal) therefore, the findings may not be entirely reflective of the scenario of TAPS in Nepal in recent years that witnessed massive changes in tobacco use pattern among adolescents. However, the following reasons have rendered the relevancy of the findings; 1) increase in tobacco smoking and smokeless tobacco use during the period 2006-2016 among the young male population (Shrestha et al., 2019), 2) history of TIs ignoring the executive orders of 1992: a ban on smoking in public places and 1998: a ban on advertising cigarette smoking in all electronic media (Bhatta et al., 2019b), and 3) WHO tobacco assessment report that highlighted Nepal government’s inability to restrict the advertisements from international TV and radio stations (Shrestha et al., 2019). The second limitation of the study is that it is a cross-sectional study, which inherently lacks the ability to claim a causal association between exposure to TAPS and cigarette smoking behaviors among adolescents. Third, the data was collected through a self-reported questionnaire, thus under reporting may have occurred, mostly among female (Shrestha et al., 2019). However, self-reported smoking behavior has been shown to be a reliable and valid method (Stein et al., 2002). Finally, the study did not account for social media marketing and the results are not generalizable to adolescents who do not go to school.

Despite these limitations, our study has major strengths. The dataset is nationally representative, which makes the results generalizable to adolescents of Nepal. It is the first study to assess the relationship between TAPS exposure and cigarette use behaviors among Nepalese adolescents, hence it provides a baseline insight into the TAPS and smoking behaviors for future research. 

The study findings show poor adherence of the Nepalese government to the world health organization’s framework convention on tobacco control (WHO FCTC) article 13, that states a comprehensive ban on TAPS. It implies active surveillance of tobacco products and future research tobacco marketing in Nepal. Also, the results highlight a need for the development of targeted interventions to reduce cigarette use initiation among Nepalese adolescents.
